# Supplementary material for: Disruption of Atrial Rhythmicity by the Air Pollutant 1,2-Naphthoquinone: Role of Beta-Adrenergic and Sensory Receptors
Source: Biomolecules. 2023 Dec 31;14(1):57. doi: 10.3390/biom14010057 (PMC10813334; doi:10.3390/biom14010057)

Supplemental figure 1. Potential pathways underlying the effects of 1,2-Naphthoquinone (1,2-NQ) on the heart. The ambient pollutant 1,2-Naphthoquinone (1,2-NQ) or its metabolites exhibits interactions with a variety of proteins and receptors, to cause complex and varied intracellular reactions. Notably, 1,2-NQ instigates lipid peroxidation, cAMP-response element binding protein (CREB) signaling, triggers the mitogen-activated protein kinase/extracellular signal-regulated kinases (MAPK/ERK) pathway through epidermal growth factor receptor (EGFR), and induces the generation of reactive oxygen species. Early exposure of mice to 1,2-NQ evokes a lasting positive chronotropic effect in adulthood, impacting TRPV1 receptors and activating the β1-adrenergic receptor. This modulation of cardiac rhythm, particularly shown in the atria, stems from the multifaceted activation of various signaling pathways by 1,2-NQ. This disruption in organism homeostasis predisposes the atria to arrhythmogenic events, whether directly or indirectly. The identification of these interconnected pathways represents a crucial advancement in our understanding of the link between air pollution exposure and atrial fibrillation. This insight sheds light on potential causal factors contributing to this cardiac condition, emphasizing the intricate molecular mechanisms through which 1,2-NQ disrupts cardiac function and ultimately poses a risk for arrhythmias.
Keys: ROS: Reactive Oxygen Species, LOOH: Lipid Hydroperoxide, NOS: Nitric Oxide Synthase, NO: Nitric Oxide, ONOO: Peroxynitrite, Ca2+: Calcium Ion, Ca2+-CAM: Calcium-Calmodulin Complex, O2-: Superoxide Anion, MAPK/ERK: Mitogen-Activated Protein Kinase/Extracellular Signal-Regulated Kinase.

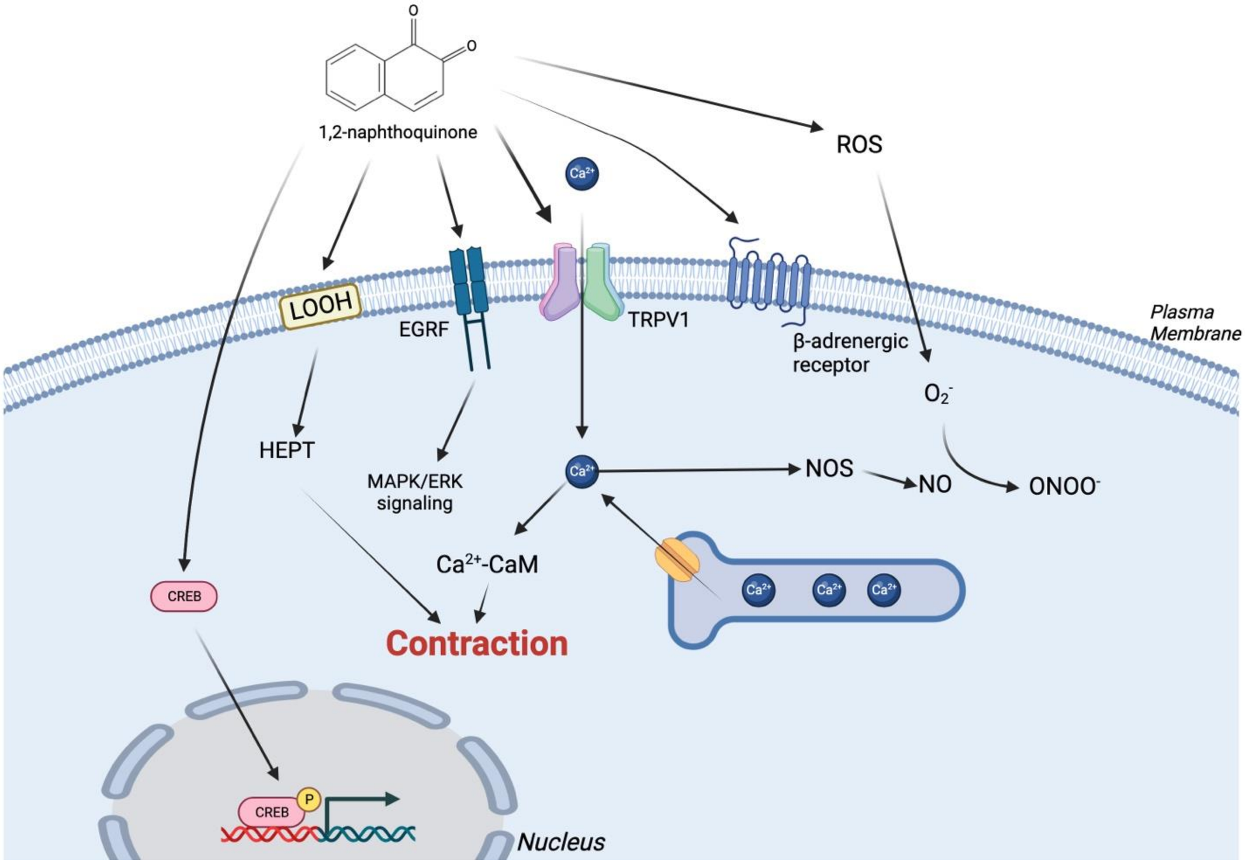

Supplement: Supplementary file 1 [file biomolecules-14-00057-s001.zip › Supplemental figure 1 - biomolecules-2756980.pdf]
