# Supplementary material for: Disruption of Atrial Rhythmicity by the Air Pollutant 1,2-Naphthoquinone: Role of Beta-Adrenergic and Sensory Receptors
Source: Biomolecules. 2023 Dec 31;14(1):57. doi: 10.3390/biom14010057 (PMC10813334; doi:10.3390/biom14010057)
Supplement: Supplementary file 1 [file biomolecules-14-00057-s001.zip › Supplemental methods_biomolecules-2756980.pdf]

Supplementary methods

Reactivity assessment of isolated right atria and the evaluation of ouabain-induced fibrillation

Mice were anaesthetised under 3% isoflurane, and an incision was made in the abdomen, displacing tissues to facilitate blood collection from the abdominal aorta. Subsequently, the chest was carefully opened, and the heart, along with the lungs, was excised and placed in a petri dish containing Kreb's solution comprising (in mM) 115 NaCl, 25 $NaHCO_3$, 4.7 KCl, 1.2 KH_2PO_4, 1.2 $MgSO_4$-$7H_2O$, 2.5 $CaCl_2$-$2H_2O$, 0.01 EDTA, and 11.1 dextrose. This solution was continuously aerated with a mixture of 95% O_2 and 5% CO_2 (v/v). This technique is rapid, ensuring the heart maintains its rhythmic beating, an essential requirement.

The heart and lungs were then transferred to a black silicon plate and secured with pins to facilitate the excision of the right atria. It is pivotal at this stage to avoid unnecessary manipulation of the tissue, aiming to make incisions as close to the heart as possible without disturbing the pacemaker cells or causing undue stretching, particularly when removing the connective tissue.

Carefully, a tungsten wire (4-5 cm) was inserted into the apical region of the atria, followed by the introduction of a second tungsten wire (4-5 cm) into the distal region of the atria. This critical step necessitates the use of ophthalmic scissors and forceps, with utmost care to prevent tissue stretching. Subsequently, the atria were moved to a wire-myograph chamber and affixed to the transducer while ensuring no stretching of the atria occurred.

Once all chambers were set up with the tissues, the temperature was regulated and maintained to 37°C ± 0.2%. A continuous aeration of 95% O_2 and 5% CO_2 was maintained. As the temperature stabilised, the system was calibrated by setting the parameters to zero and gradually applying tension to the tissues. Simultaneously, the heart's beating signal was monitored using LabChart Pro (AdInstruments) or a similar data acquisition programme. The tissues were delicately stretched until a tension of approximately 2.50 mN was achieved. Typically, the peaks of beating atria fall within the range of 0.5-1.0 mN.

Upon setting the tension and achieving stable temperature, the tissues underwent a washing process at 15-minute intervals over a span of 1 hour, with tension adjustments made as necessary. After an hour equilibration, the tissues were prepared for experimentation.

We utilised a DMT620 Wire-Myograph (AdInstruments), necessitating adjustments in line with the manufacturer's manual recommendations before the calibration process. These settings included: - Force recording output range – 50 mN; Measurement range – 200 mN; and Sample Rate - 1 k/s. It is advisable to configure these adjustments before

mounting the tissue in the myograph chamber or, preferably, the day prior, saving the settings as a file in LabChart Pro. The data acquired were directly analysed using the LabChart Pro Module "Peak Analysis," pre-installed, and expressed as beats per minute (BPM).

Finally, concentration-response curves were assessed by conducting experiments to evaluate positive and negative chronotropism using norepinephrine (NE) and carbachol (CCh), respectively. Pre-prepared aliquots of both drugs were used (10-3 - 10-8 M). Following stabilization, NE was added incrementally to achieve final concentrations ranging from 10^{-10} to 10^{-6} M, with a 5-minute interval between doses. Once the NE concentration-response assay was completed, tissues were washed with Kreb's solution for 15 minutes. After stabilization, CCh was added at final concentrations ranging from 10^{-10} to 10^{-6} M, again with a 5-minute interval between doses.

For the assessment of ouabain-induced fibrillation, the same protocol was followed until step 9. Subsequently, the atria were incubated with ouabain (10 μM) for 30 minutes. The addition of this drug induced irregular contractions after 30 minutes. We then could evaluate both the force of contraction (mN) recorded and the time taken for the onset of irregular contractions. When the atria were incubated with both ouabain and 1,2-NQ (100 nM), the irregular contraction duration increased, enhancing the contraction force, and reduced the onset time of irregular contractions.
